# Effect of Alkaline Salts on Calcium Sulfoaluminate Cement Hydration

**DOI:** 10.3390/molecules26071938

**Published:** 2021-03-30

**Authors:** Luís Urbano D. Tambara Júnior, Janaíde C. Rocha, Malik Cheriaf, Pilar Padilla-Encinas, Ana Fernández-Jiménez, Angel Palomo

**Affiliations:** 1 Laboratory of Waste Valorization and Sustainable Materials (ValoRes), Department of Civil Engineering, Federal University of Santa Catarina (UFSC), Florianópolis 88040900, Brazil; janaide.rocha@ufsc.br (J.C.R.); malik.cheriaf@gmail.com (M.C.); 2 Eduardo Torroja Institute, National Research Council (CSIC), 28033 Madrid, Spain; maria.padilla@ietcc.csic.es (P.P.-E.); anafj@ietcc.csic.es (A.F.-J.)

**Keywords:** calcium sulfoaluminate cement, alkaline salts, microstructure, ettringite

## Abstract

This work analyzes the effect of the presence of 5 wt.% of solid sodium salts (Na_2_SO_4_, Na_2_CO_3_, and Na_2_SiO_3_) on calcium sulfoaluminate cement (CSA) hydration, addresses hydration kinetics; 2-, 28-, and 90-d mechanical strength, and reaction product microstructure (with X-ray diffraction (XRD), and Fourier transform infrared spectroscopy, (FTIR). The findings show that the anions affect primarily the reactions involved. Ettringite and AH_3_, are the majority hydration products, while monosulfates are absent in all of the samples. All three salts hasten CSA hydration and raise the amount of ettringite formed. Na_2_SO_4_ induces cracking in the ≥28-d pastes due to post-hardening gypsum and ettringite formation from the excess SO_4_^2–^ present. Anhydrite dissolves more rapidly in the presence of Na_2_CO_3_, prompting carbonation. Na_2_SiO_3_ raises compressive strength and exhibits strätlingite as one of its reaction products.

## 1. Introduction

Due primarily to the immensity of worldwide output, Portland cement (PC) manufacture is deemed a major source of greenhouse gases, in particular CO_2,_ prompting the scientific community and the industry to develop and use alternative cements. Calcium sulfoaluminate (CSA) cement is more eco-friendly than PC, inasmuch as CSA clinker contains less calcium, requires calcination temperatures around 200 °C lower and is more readily ground, factors that together translate into lower energy consumption and CO_2_ emissions [1].

CSA first began to be industrially developed in China in the nineteen seventies. Its foremost mineral phases include ye’elimite (C_4_A_3_$) and belite (C_2_S), with smaller contents of ferrite (C_4_AF), calcium aluminate (CA), and calcium sulfate (C$). That third mineral is also added during grinding in the form of gypsum or anhydrite [2,3,4]. CSA exhibits high early-age mechanical strength, although the hydration products formed, which differ from the ettringite and AH_3_ (Equation (1)) [5,6,7] found in PC, might cause durability problems at later ages.

Manufacture has spread to other countries in recent years, where CSA is often used as a component of blended cements for applications requiring high early-age strength [8].

C_4_A_3_$ + 2 C$H_x_ + (38 − 2x) H → C_6_A$_3_H_32_ + 2 AH_3_ x = 0, 0.5, 2 (ettringite formation) (1)

According to some studies, when blended with PC, calcium sulfoaluminate cement may raise early-age mechanical strength, reduce shrinkage, control setting time, and improve frost resistance [9,10,11,12]. In CSA + PC blends, the calcium hydroxide released during PC hydration combines with C_4_A_3_$ and calcium sulfate to generate microcrystalline ettringite, which largely offsets shrinkage. The pore size distribution in CSA + PC blends is skewed toward the submicron range [13,14]. Earlier setting times and earlier strength development have also been observed in CSA/PC/calcium aluminate cement (CAC) ternary systems, along with strength growth at later ages due primarily to the low porosity of these materials [15].

Another possibility, scantly explored to date, is to formulate new CSA blends with what are known as alkaline cements (HYC) [16,17]. The latter are characterized by high proportions of additions or cement replacements (usually fly ash (FA), blast furnace slag, or calcined clay) and a low PC content (<30%). Whilst either liquid or solid alkalis may be added to catalyze the initial reaction in the replacements [18], the use of solids, such as Na_2_CO_3_, Na_2_SO_4_, or Na_2_SiO_3_, are able to react with the portlandite forming during PC hydration, generating caustic soda in situ, is much more practical [19,20,21,22].

The use of CSA blended with hybrid alkaline cements to raise early-age strength entails an understanding of how the presence of such salts may affect hydration and mechanical strength development. Prior studies [23] have shown the presence of Li_2_CO_3_ in ternary systems (CSA/CP/anhydrite) to hasten ye’elimite and C_3_S dissolution and with it ettringite precipitation. Excess Li_2_CO_3_ modified the Ca/Al ratio of the aqueous phase, however, as well as the hydration reactions, in which the CSA hydration initially prevalent (aluminium hydroxide, ettringite, and strätlingite precipitation) was followed by a predominance of PC hydration, with C-S-H and ettringite formation. Na_2_SO_4_, in turn, proved able to form strätlingite (a phase stable in high aluminosilicate systems but which decomposes in the presence of portlandite) in CAC/silica fume blends due to the action of sodium ions [24].

The present study aimed to determine the effect of three sodium salts (Na_2_SO_4_, Na_2_CO_3_, and Na_2_Si_2_O_5_·2H_2_O) on the hydration of a commercial CSA. More specifically, it explored how these salts impact setting, mechanical strength development over time, and the nature of the reaction products formed. 

## 2. Results

### 2.1. Isothermal Conduction Calorimetry

**Figure 1:**
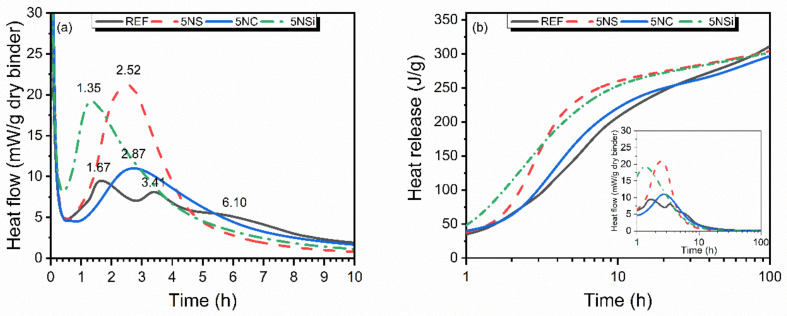
(**a**) Heat flow and (**b**) total heat released and detail of heat flow curve.

The effect of the salts on heat flow and total heat released during CSA hydration is illustrated in Figure 1a) and Table 1. The heat flow curve for reference (REF) exhibited a number of peaks, the first beginning with initial contact between the material and the water, although the instrument recorded only the downward slope of that peak. The second peak reached a maximum at 1.67 h and the third at 3.41 h, with a shoulder appearing after 5 h of hydration. The pastes containing alkaline salts exhibited a single peak on the heat flow curves, which was more intense than any observed for REF, especially when the salt was Na_2_SO_4_. The induction period was delayed slightly with Na_2_CO_3_, remained unchanged with Na_2_SO_4_ and was shortened with sodium disilicate. 

Total heat release for the four systems is graphed in Figure 1b). The presence of any of the three salts raised the heat initially released, although the values tended to converge at around 300 J/g after 90 h. The higher initial value is consistent with the greater intensity of the peaks on the heat flow curve.

### 2.2. Mechanical Strength, Consistency, and Setting Times

The presence of salts affected CSA paste flowability. Further to the mini-slump test results, CSA paste was less fluid in the presence of Na_2_CO_3_ (68.5 mm) and Na_2_SiO_3_ (95.25 mm) than in the reference paste (103.5 mm) and more fluid with Na_2_SO_4_ (115 mm), although all four materials exhibited suitable workability.

**Figure 2:**
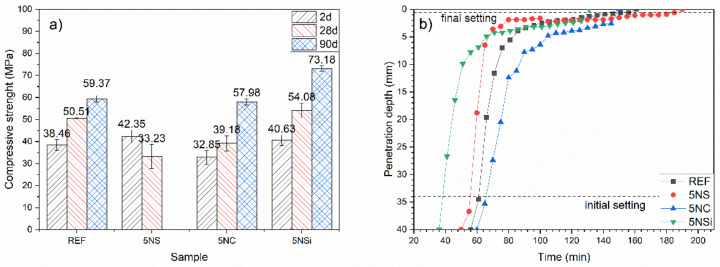
(**a**) Compressive strength and (**b**) setting times for 2-, 28-, and 90-d pastes.

Two-day, 28-d, and 90-d compressive strength is graphed in Figure 2a). The 2-d strength found for the water-hydrated CSA (REF), 38 to 46 MPa, grew with time, reaching 59.37 MPa in the 90-d cement. Whilst the use of 5% Na_2_SO_4_ (5NS) in place of cement raised 2-d compressive strength relative to the reference, the 28-d specimens exhibited surface cracks and a decline in strength values. The 90-d specimens were too cracked to test for compressive strength (see Supplementary Material Figure S1). 

The compressive strength observed in paste 5NC (containing Na_2_CO_3_) was slightly lower than in the reference (REF) at all ages studied. Although these specimens were not cracked, they exhibited efflorescence (see Figure S1), which when analyzed with Fourier transform infrared spectroscopy, (FTIR) (see Figure S2) was found to be due to sodium sulfate and calcium carbonate formation. 

The presence of sodium silicate (5NSi) translated into higher strength than the reference (REF) at all of the ages studied. Strength gain with time grew by around 20 MPa between the 28- and 90-d specimens, compared to just under 10 MPa in the reference CSA. 

According to the initial and final setting times plotted in Figure 2b), REF exhibited initial setting (*t_i_*) after 1 h and final setting (*t_f_*) after 2 h 21 min. Initial setting came very early in 5NC, which broke and had to be remixed, a behaviour associated with false setting. Otherwise, as Figure 2b) shows, this paste had setting times very similar to those observed for REF (*t_i_* = 1 h 6 min; *t_f_* = 2 h 30 min). Paste 5NS also exhibited times (*t_i_* = 56 min; *t_f_ =* 2 h 10 min) resembling the REF values, whilst initial and final setting times were shorter in paste 5NSi, at 38 min and 1 h 30 min, respectively. Vicat apparatus-determined initial setting was fairly consistent with the beginning of the heat flow curve peaks (Figure 1), according to which acceleration had a shorter duration in 5NSi. Final setting time was very close to the maximum variation in heat flow in all but REF, in which final setting time appeared at mid-downslope of the second peak.

### 2.3. Characterization

**Figure 3:**
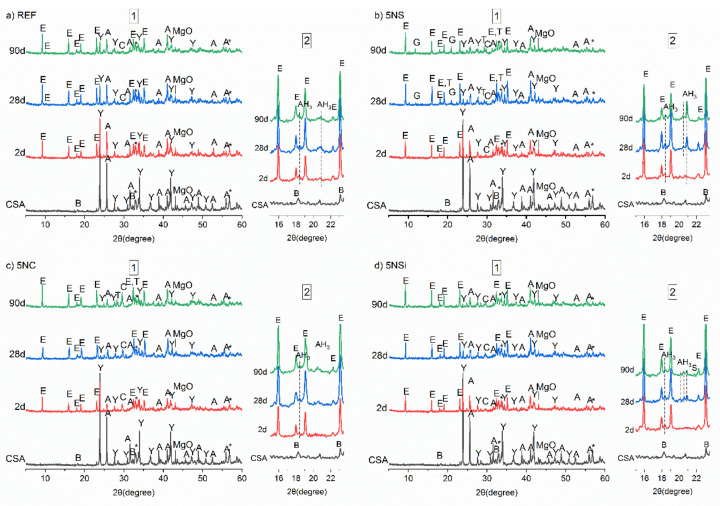
1—Full diffractograms; 2—detail of patterns across 2θ 16° to 22° for anhydrous calcium sulfoaluminate cement (CSA) and pastes: (**a**) reference (REF); (**b**) 5% Na_2_SO_4_ (5NS); (**c**) 5NC; (**d**) sodium silicate (5NSi); legend: A—anhydrite; Y—ye’elimite; E—ettringite; B—belite, bredigite; T—thenardite; C—calcite; G—gypsum; AH_3_—aluminum hydroxide; S—strätlingite (C_2_ASH_8_).

The X-ray diffraction (XRD) patterns for anhydrous CSA and 2-, 28-, and 90-d water-hydrated pastes in the absence (REF) and presence (5NS, 5NC, and 5NSi) of alkaline salts are reproduced in Figure 3. The main diffraction lines on the diffractogram for water-hydrated CSA paste (REF) matched the reflections for ettringite and the unreacted phases C_4_A_3_$ and C$, which declined in intensity with hydration time. The many weak reflections at 18° to 19° and 20° to 22°, which rose in intensity over time, were indicative of the formation of scantly crystalline AH_3_ [25] (see detail in Figure 3a). No AFm-like phases were identified. 

The most intense reflections on the diffractograms for the pastes hydrated in the presence of salts were related to ettringite formation. The intensity of the signals associated with ye’elimite was apparently lower than on the patterns for REF pastes. In the presence of Na_2_SO_4_ (Figure 3b), 5NS pastes, in addition to ettringite, AH_3_, gypsum, and thenardite were observed to form in the 28-d specimens, with the respective reflections rising in intensity in the 90-d pastes. The intensity of the line for ettringite declined slightly between 28 and 90 d in sample 5NS, whilst no AFm was observed to form. The AH_3_ in this paste exhibited greater crystallinity than in the reference. 

In the 5NC pastes (bearing Na_2_CO_3_), the lower intensity of the anhydrite reflections than in REF at all three ages was associated with greater phase dissolution. No gypsum was observed to form, although calcite was present in the ≥2-d pastes and thenardite in the 90-d specimens. The signals associated with AH_3_ formation were scantly detectable in the pastes hydrated in the presence of this salt. 

The 5NSi pastes exhibited the same products as in the reference, namely ettringite, AH_3_, and calcite, although the 90-d reflections for calcite and ettringite were slightly more intense. Strätlingite (C_2_ASH_8_), not identified with XRD in any of the other pastes, was also observed to form, with low intensity reflections at 12.6° and 21.3° (see detailed in Figure 3d).

### 2.4. Microstructural Analysis, FTIR

**Figure 4:**
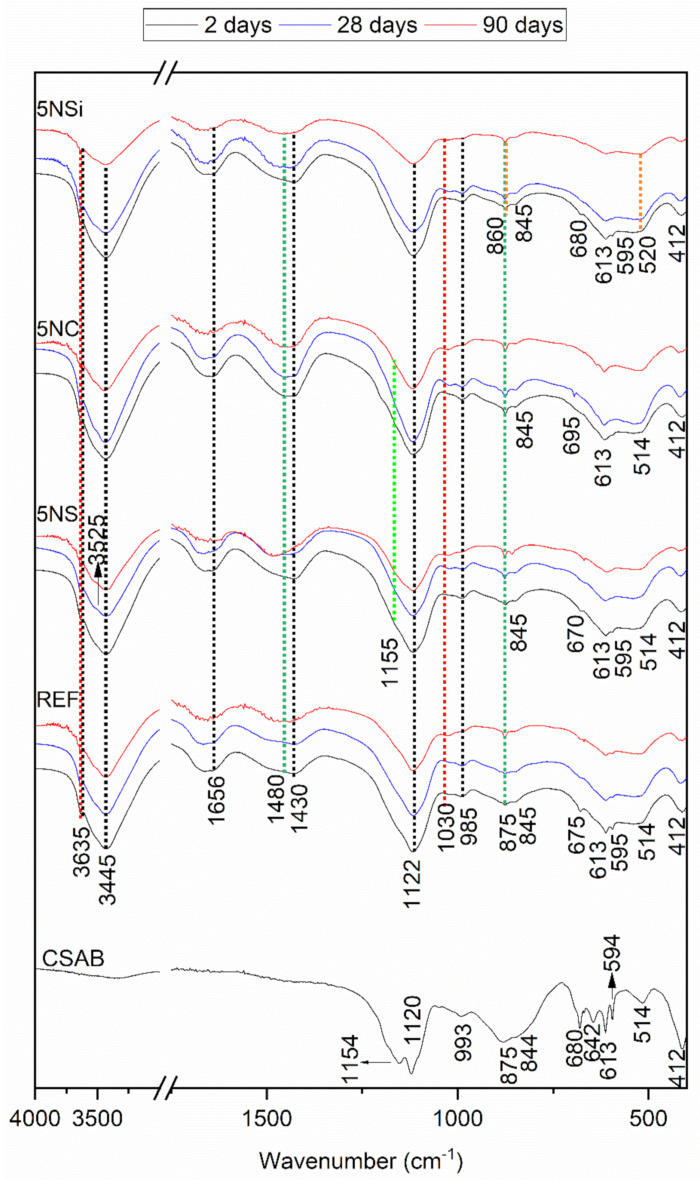
Two-day, 28-d and 90-d Fourier transform infrared spectroscopy, (FTIR) spectra for REF, 5NS, 5NC, and 5NSi pastes; legend: red line—AH_3_, black line—ettringite, green line—calcite, blue line: thenardite, orange line—strätlingite.

The FTIR spectra for the anhydrous CSA and all of the 2-, 28-, and 90-d pastes studied, reproduced in Figure 4, confirmed and supplemented the XRD findings. The main bands on the anhydrous CSA spectrum were associated with asymmetric stretching vibrations at 1190 cm^−1^ and 1100 cm^−1^ attributable to SO_4_^2−^, along with υ_1_ stretching bands at 875 cm^−1^ and 812 cm^−1^, indicative of the AlO_4_ present in ye’elimite [26]. Signals associated with the presence of anhydrite, generated by υ_3_ SO_4_ were visible at 1175 cm^−1^, 1150 cm^−1^, 1120 cm^−1^ and 1048 cm^−1^ and with υ_1_ SO_4_ at 680 cm^−1^, 613 cm^−1^ and 594 cm^−1^. The presence of C_2_S in CSA was inferred by the υ_3_ Si-O vibrations at 999 cm^−1^ and 911 cm^−1^, υ_1_ Si-O vibrations at 845 cm^−1^ and υ_4_ Si-O vibrations at 514 cm^−1^ [27]. 

The spectra for the pastes clearly exhibited bands associated with vibration frequencies at: 3635–3637 cm^−1^ and 3440 cm^−1^, indicative of OH-stretching in AH_3_ (a phase difficult to detect with XRD, given its low crystallinity); 1025 cm^−1^, of Al-O-H bending; and 854 cm^−1^, of AlO_4_ stretching. Some of the AH_3_ bands (at 3635 cm^−1^ and 3440 cm^−1^) overlapped with signals associated with OH stretching or (at 1656 cm^−1^) with OH bending in ettringite, whereas another band at 1115 cm^−1^ was indicative of AlO_4_ asymmetric stretching [28].

The 28- and 90-d spectra for the pastes hydrated with 5NS contained bands: attributed to gypsum; at 3520 cm^−1^ a band associated with OH bending vibrations in water; one at 1480 cm^−1^ generated by S-O stretching; and one at 856 cm^−1^ by SO_4_ tetrahedron bending [29] (data confirmed by XRD). Thenardite was identified on the grounds of the SO_4_ asymmetric stretching bands at 613 cm^−1^ and 1155 cm^−1^ [30].

The highest intensity bands on the 5NC spectrum were associated with carbonates in the form of calcite (1450 cm^−1^ and 875 cm^−1^) [31]. Nahcolite (NaHCO_3_) was visible primarily in the 28-d specimens, identified as absorption bands at 1646 cm^−1^ and 1630 cm^−1^ (O-H bending) and 695 cm^−1^ (HCO_3_ group bonds) [32]. The 5NSi spectra exhibited the same hydration products as the reference paste, while at later ages the presence of vibration bands associated with strätlingite formation were also detected [33]. 

#### Deconvolution of FTIR Spectra in the 1350 cm^−1^ to 700 cm^−1^ Range

**Figure 5:**
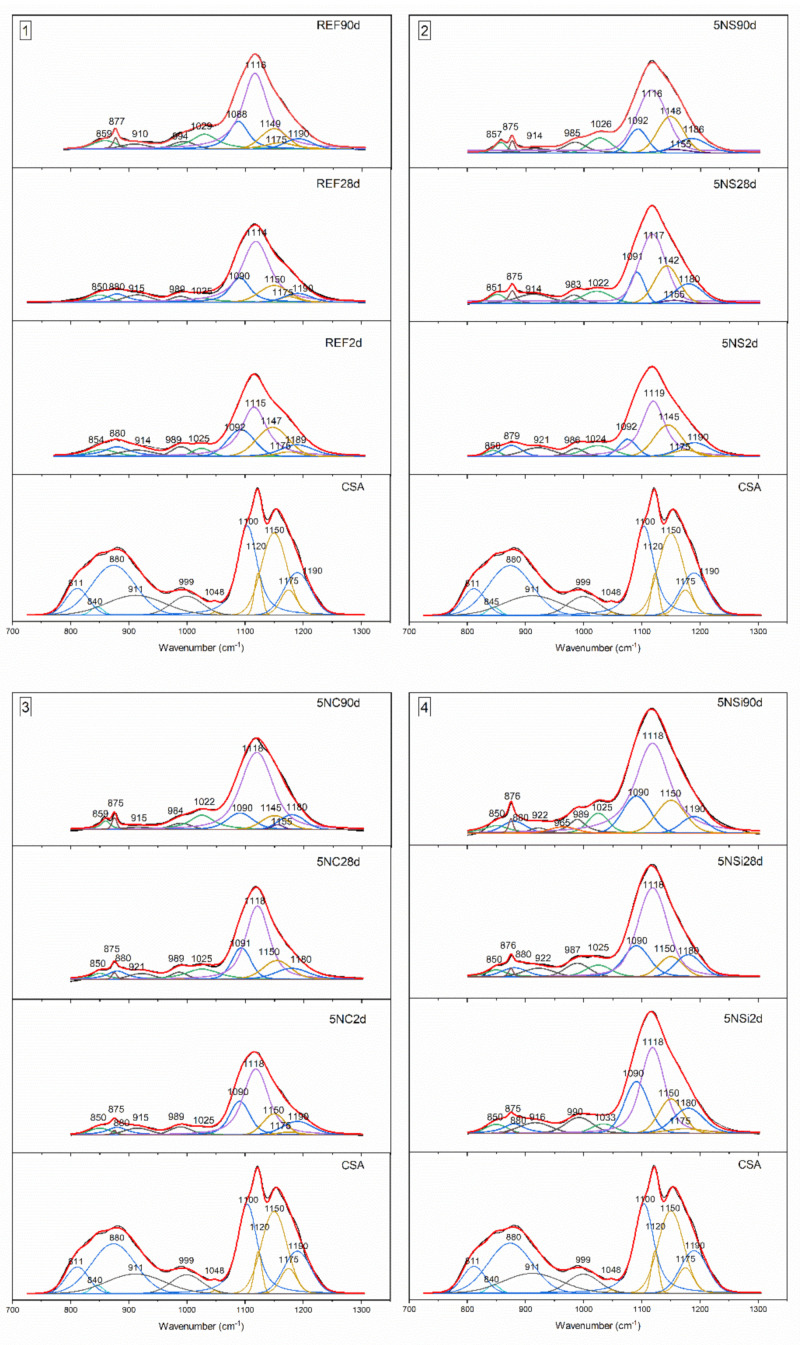
Deconvoluted 2-, 28-, and 90-d spectra (1350 to 700 cm^−1^ range) for CSA hydrated with water and alkaline salts; legend: blue: —/ye’elimite; yellow—C$; grey—C_2_S; green—AH_3_; violet -ettringite; brown—CaCO_3_; burgundy—strätlingite; purple—thenardite; lime—C_3_A, red—cumulative fit.

Given the overlapping observed and the concomitant complexity involved in differentiating bands, a detailed study was conducted of the 1350 cm^−1^ to 700 cm^−1^ spectral range using the Voigt function. Regression coefficients (r^2^) varied from 0.99996 to 0.99921. The band positions resulting from second derivative analysis are shown in Figure 5, while the positions and respective areas are listed in Table 2. 

The deconvoluted spectrum for anhydrous CSA contained 11 signals (see Table 2) associated with the phases in the original material, all of which declined in intensity with hydration time. Some of the bands associated with phases indicative of AlO_4_ stretching (811 and 845 cm^−1^) and SO_4_^2−^ asymmetric stretching (1048 and 1120 cm^−1^) were absent even in in the 2-d spectra for all of the mixes, whilst the signal at 1175 cm^−1^ declined more gradually over time. 

Similar bands were observed in the spectra for all of the pastes at: 1115 to 1119 cm^−1^, indicative of the SO_4_^2−^ asymmetric stretching in ettringite [28]; 850 to 859 cm^−1^, of AlO_4_ stretching; and 1022 to 1023 cm^−1^, of Al-O-H bending in AH_3_. The band at 1150 cm^−1^ on the spectrum for 5NS, associated with gypsum formation, rose in the 28- and 90-d specimens. The band at 1155 cm^−1^ on the 5NS and 5NC spectra was indicative of thenardite formation. The area under the ettringite band (~1116 cm^−1^) increased in keeping with hydration time, except in 5NS, where the area declined slightly from 34.88% in the 28-d to 32.87% in the 90-d specimens. The 90-d spectra for the 5NSi pastes exhibited a band at 965 cm^−1^ associated with strätlingite formation [33]. 

### 2.5. Scanning Electron Microscope (SEM) 

**Figure 6:**
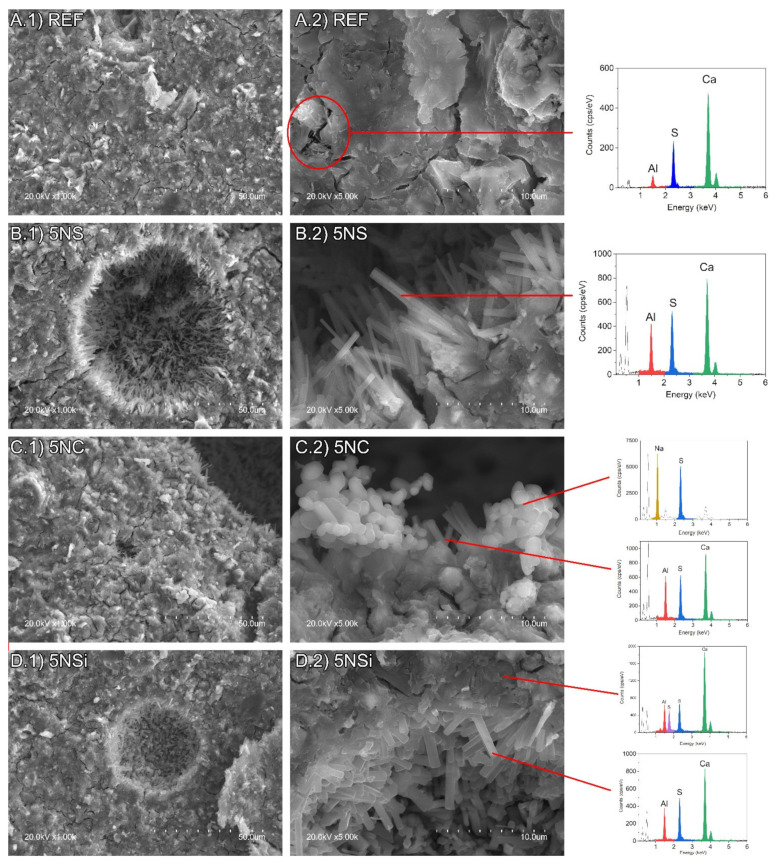
Scanning electron microscope (SEM) micrographs (at 1000× and 5000×) of 28-d: (**A**) REF; (**B**) 5NS; (**C**) 5NC; and (**D**) 5NSi.

**Figure 7:**
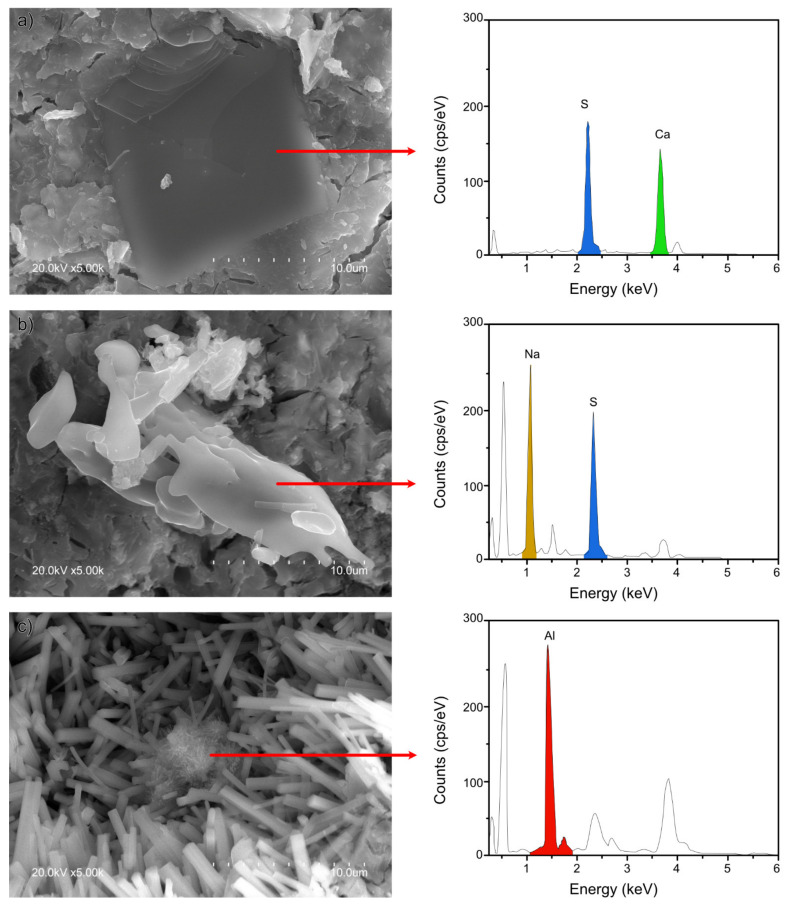
SEM micrographs (at 5000×) of sample 5NS showing: (**a**) gypsum; (**b**) thenardite; and (**c**) AH_3_.

The 28-d micrographs for REF, 5NC, 5NS, and 5NSi are reproduced in Figure 6. All of the pastes exhibited a dense and generally amorphous microstructure. That type of morphology, identified by Gastaldi et al. [34], is associated with CSA pastes hydrated with low water/cement ratios such as those used in this study. The presence of crystalline ettringite needles was detected in the paste pores, most abundantly in the 5NS materials (Figure 6B.2). Gypsum (Figure 7a), sodium sulfates (Figure 7b) and AH_3_ microcrystals (Figure 7c) were also observed to form in 5NS. Cracking in these matrices was attributable primarily to the presence of gypsum prismatic and thenardite clustered crystals (see Energy Dispersive X-Ray Spectroscopy (EDS) analysis in Figure 7). Thenardite was also identified in paste 5NC (Figure 6C.2), which exhibited the shortest ettringite needles. 

**Figure 8:**
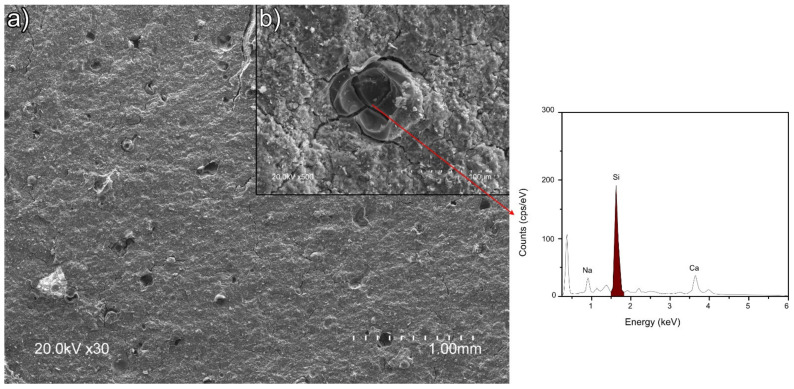
SEM micrograph of sample 5NSi showing: (**a**) porous zones and (**b**) enlargement of insoluble silicate.

The size of the ettringite crystals in sample 5NSi was similar to the dimensions observed for REF. No strätlingite-like particles (identified only after 90 d by XRD and FTIR) were detected on the 28-d micrographs, inasmuch as that mineral forms more slowly [35]. EDS analysis of the amorphous matrix revealed the presence of silicon (see Figure 6D.2), a Ca/Si ratio of 0.15 to 0.35 and an Al/Ca ratio of around 1, the theoretical value. Unreacted, low solubility (11.5 g/100 mL [36]) sodium silicate was observed in the 28-d micrographs (Figure 8). The qualitative SEM-EDS analyses identified phases with similar morphologies and compositions. Further to the data, the Ca/S ratios for the ettringite needles were slightly higher than the theoretical ratio of 2 (~2.38 for REF; 2.18 for 5NS; 2.34 for 5NC; and 2.63 for 5NSi). 

## 3. Discussion

Further to Equation (1), for ye’elimite hydrated in the presence of anhydrite to yield ettringite, the water/CSA (w/c) ratio would need to be 0.78 (Equation (2). The CSA used in this study contained around 70% C_4_A_3_$ + C$, inferring that the w/c ratio used here should have been the stoichiometric 0.54. That notwithstanding and in keeping with actual practice, a slightly lower ratio (w/c = 0.5) was applied, although the resulting pastes were workable. That lower amount of water than stoichiometrically required delivered denser matrices and less crystalline hydration products [37], as observed in Figure 6, Figure 7 and Figure 8.

(w/s)st = gr H_2_O/gr (C_4_A_3_$ + C$) = (34 × 18)/(610 + 2 × 136.14) = 0.78 (2)

The heat flow curve for CSA hydration (REF, see Figure 1) contained a signal peaking at 1.67 h, according to Jensen et al. [38] a time associated with synchronized C_4_A_3_$ + C$ reaction-induced ettringite and amorphous AH_x_ (AH_3_ with a higher water content) formation and precipitation. The two following peaks, at 3.41 and 5 h, would be indicative of more ettringite precipitation, which would dissolve no more calcium sulfate nor consume unbound water. Rather, the water for ettringite precipitation would be sourced from the excess water in the amorphous AH_x_ forming. The presence of AFt and AH_3_ was verified by XRD and FTIR characterization of the 2-d pastes, which exhibited some minor carbonation in the form of calcite (see Figure 3), observed to increase in intensity over time. 

The presence of salts modified the heat flow curve. A single peak, instead of the three observed in REF, appeared at different times and with a higher intensity, suggesting that the salts may have induced simultaneous coursing of some of the reactions involved. Similar behaviour was found in an earlier study analyzing the effect of pH on CSA hydration [17], where ~0.1 to ~1 M NaOH solutions hastened the initial CSA reaction, raising the initial heat of hydration and early-age strength. That behaviour differed at pH > 14. In addition to the effect of the presence of alkalis (pH < 12), the present study analyzed the effect of anions SO_4_^2−^, CO_3_^2−^, and SiO_4_^2−^. 

**Figure 9:**
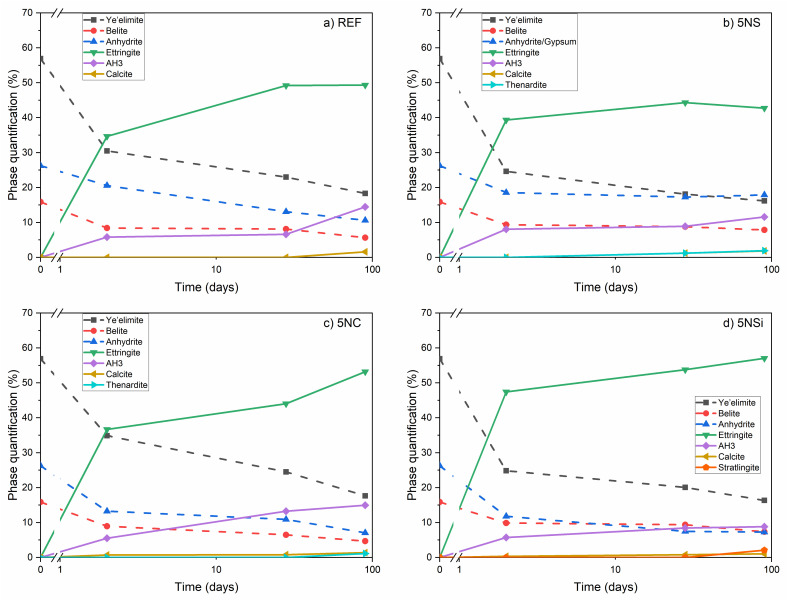
Phase content found by deconvoluting FTIR spectra for: (**a**) REF; (**b**) 5NS; (**c**) 5NC; and (**d**) 5NSi.

Figure 9 compares the areas associated with the deconvoluted FTIR bands in the 1350 to 700 cm^−1^ range on the spectra for the 2-, 28-, and 90-d pastes. The pastes hydrated in the presence of the salts were observed to consume more anhydrite and ye’elemite. The 2-d belite content declined slightly, but remained essentially flat thereafter. The effect of each salt is discussed in greater detail below. 

### 3.1. Hydration in the Presence of Na_2_SO_4_

Sodium sulfate may cause severe durability problems in conventional Portland cements [39,40]. In contrast, the use of sodium silicate at concentrations of 5% to 8% in hybrid cements (~70% to 80% fly ash and 30% to 20% PC) has yielded promising results [41]. The data in Figure 1 and Figure 2 show that in CSA cements 5% Na_2_SO_4_ hastened initial hydration and improved 2-d compressive strength, while inducing material cracking and a decline in strength at later ages (see Figure S1).

As Na_2_SO_4_ is a very soluble salt (~20 g/100 mL of water at 20 °C), only traces of its presence were detected by XRD or FTIR in 2-d 5NS pastes. The presence of a higher sulfate ion content appeared to hasten the initial ye’elimite reaction, giving rise to the formation of more ettringite and AH_3_ gel in the 2-d pastes. That would explain the higher 2-d strength values (Figure 2), as well as the presence of an intense peak on the heat flow curve (Figure 1). Cracking appeared between the 2- and 28-d 5NS specimens (see Figure S1b), whilst the XRD and FTIR spectra revealed an ongoing decline in ye’elimite content, a rise in the proportion of ettringite and the onset of sulfate salts (gypsum and thenardite). The cracks appearing at 28 d and especially at 90 d were associated with the internal stresses generated in the specimens. Due to the precipitation of these salts in the hardened matrix (Figure 7), the process was intensified in the 90-d material by the slight decline in ettringite content (Figure 9b). The products identified were similar to the ones found when PC, attacked by sulfates, cracks in response to their formation [42,43]. 

The aforementioned developments were exacerbated by carbonation, in turn favoured by the higher pH generated by the presence of alkalis. That affected AFt stability and induced gypsum formation, as per Equation (3) [44].

3CaO∙Al_2_O_3_∙3CaSO_4_∙32H_2_O + 3CO_2_ → 3CaSO_4_∙2H_2_O + 3CaCO_3_ + 2Al(OH)_3_ + 23H_2_O (3)

Figure 9b clearly shows that ettringite content declined between 28 and 90 d, while the gypsum, calcite, and AH_3_ contents rose. There was more AH_3_ in paste 5NS than in REF (CSA + water). The XRD and SEM findings (Figure 3 and Figure 6) also revealed gibbsite formation, an indication of greater AH_3_ crystallinity. Gibbsite crystal growth may have been related to the increase in voids generated by the cracks induced by ettringite and gypsum formation. 

The change in hydrate content observed for 5NS was associated with the AFt stability domain in CaO∙Al_2_O_3_∙Ca_2_SO_4_∙H_2_O systems. Rising sulfate concentration modifies the stability field of ettringite [45], inducing its decomposition along with the formation of gypsum, calcite, and thenardite [46,47,48]. Such ettringite destabilization, together with what is known as ‘sulfate salt crystallization distress’, prompts variations in volume and material cracking (see Figure S1) [40,47]. 

In contrast, the presence of 5% Na_2_SO_4_ appeared to have no significant impact on the degree of C_2_S hydration. That result is consistent with other studies published in the literature [49] on the effect of 4% sodium sulfate on synthetic C_2_S hydration. 

### 3.2. Hydration in the Presence of Na_2_CO_3_

The Na_2_CO_3_ content (ranging from 2 to 4 wt.%) in PC may induce carbonation and efflorescence [50]. In hybrid cements it is used to catalyze hydration and raise early-age strength [51,52]_._ This highly soluble salt (30.7 g/100 mL) also raises pH to values of ~11.5. Its presence during CSA hydration appeared to have no significant effect on that reaction at either early or later ages. Setting times and strength development in the specimens studied here were very similar to the findings for the reference paste (REF = CSA + water), although more calcite and surface efflorescence were observed (see Figure 9). Surface efflorescence poses aesthetic problems but apparently causes no structural damage to the material. As it consisted primarily of sodium or calcium carbonate, its presence was tantamount to releasing the salt added, thereby mitigating the potential effect of the activator and generating matrices that resembled the reference more closely than the other two salts. 

These pastes did have a higher calcite content than the reference (Figure 3 and Figure 5), however, visible at 29.5° on the XRD patterns, as the deconvoluted FTIR band at 875 cm^−1^ as well as the band at 1450 cm^−1^. Thenardite also precipitated in these matrices at later ages (90 d), further to the reaction described in Equation (4) [53].

(4) $${\mathrm{CaSO}}_{4}+{\mathrm{Na}}_{2}{\mathrm{CO}}_{3}\;\to {\;\mathrm{Na}}_{2}{\mathrm{SO}}_{4}+{\mathrm{CaCO}}_{3}$$

The carbonates added formed calcite or soluble calcium salts but appeared not to react with ettringite to any significant extent (Equation (3)), for the latter was not observed to decline or form gypsum. On the contrary, the amount of ettringite continued to rise over time (Figure 9c), which would explain the steady rise in mechanical strength (Figure 2a). Follow-up studies on developments in >90-d specimens would be advisable. 

### 3.3. Hydration in the Presence of Sodium Disilicate

At 11.5 g/100 mL, solubility was lower in Na_2_SiO_3_ than in the other salts studied here [36]. This salt raised the (highly reactive) SiO_2_ content in the overall cement composition by nearly 2%. The 90-d XRD patterns exhibited strätlingite (C_2_ASH_8_), a product not identified in the other two mixes, whose precipitation was largely induced by the higher silica content in the binder. 

Siliceous hydrogarnet (C_3_ASH_4_), observed to form in calcium aluminate cements (CAC) blended with sodium silicate, retards CAC hydration slightly [54]. In the presence of reactive silica (silica fume), however, CAC hydration has been reported to generate strätlingite (C_2_ASH_8_) [24,55,56], a reaction expedited by the presence of alkaline ions that favour silica fume dissolution. Later-age belite/ye’elimite/ferrite (BYF) cement pastes and BYF-FA blended cements have also been reported to form strätlingite and siliceous hydrogarnet. The presence of strätlingite in CSA/sodium silicate blends in this study was associated primarily with the reaction of the soluble Si ions in the silicate with the AH_3_ in the presence of calcium. The sodium silicate-induced rise in the degree of ye’elimite reaction and ettringite content and the decline in AH_3_ content, all relative to the reference, are illustrated in Figure 9d). Those data confirm reports in the literature to the effect that strätlingite formation entails the uptake of AH_3_ [57].

## 4. Materials and Methods

A commercial cement, ALICEM i-tech^©^, was used in this study. According to the particle size distribution determined on a COULTER LS130 analyzer covering a range of 0.1 to 900.0 µm, the diameter of 90% of the particles was under 11.51 µm and 10% <1.06 µm. Cement density was 2.90 g/cm^3^, its Blaine fineness 474.6 m^2^/kg and its BET specific surface 1373 ± 13 m^2^/kg (see Table 3). 

Table 3 also shows the chemical composition of the CSA established on a Bruker S8 Tiger X-ray fluorescence spectrometer fitted with an Rh-anode X-ray tube, as well as its XRD-defined mineralogical composition (interpreted with the Rietveld method using Topas [58] software and COD data). Ye’elimite accounted for 49.4% of the total, anhydrite (possibly a result of gypsum water loss during joint milling) for 23%, calcium silicate phases for 18.5% and tricalcium aluminate for 1.8%.

The CSA cement was hydrated with water in the absence (REF) or presence (5 wt.%) of one of three alkaline salts: sodium sulfate (Panreac 99% reagent grade Na_2_SO_4_), (Silicates Manufacturing Company, Madrid, Spain) sodium disilicate powder (with a SiO_2_/Na_2_O molar ratio of approximately 2.00 and a solids content of 82%) and sodium carbonate (Panreac 99.8% reagent grade Na_2_CO_3_). Mixes containing 95 g of CSA and 5 g of salt (see Table 4) were dry-blended for 30 min in a shaker-mixer and subsequently hydrated with water at a w/b ratio of 0.5, the proportion of water required to hydrate CSA further to the consistency test described in European and British standard BS EN 196-3 [59]. A low w/b ratio is recommended for CSA hydration to control porosity and limit the diffusion of aggressive agents such as carbonates and chlorides to enhance durability [34]. The weight percentages of Na_2_O and the anions SO_3_^2−^, CO_3_^2−^, and SiO_3_^2−^ are listed in Table 4. The CSA + salt + water mixes were stirred automatically at 350 rpm for 3 min.

Setting times were determined with an automatic Vicat apparatus further to EN 196-3 [59] specifications. The initial setting was defined as the time when the plunger penetrated 34 mm into the sample and the final setting as when penetration declined to 0.5 mm.

The effect of the alkaline solution on paste fluidity, determined with the mini-slump test [60], was defined as the mean of four slump diameter readings using a mould proportional in size to the standard cone (small mould/standard cone = 0.19). Mechanical strength was found on prismatic specimens (1 × 1 × 6 cm) [61], initially cured in their moulds in a climatic chamber (22 ± 2 °C, relative humidity >90%) for 24 h, when they were removed from the moulds and stored in the chamber until the test age (2, 28, or 90 d). 

Twelve samples were tested for compressive strength on an Ibertest Autotest-200/10-SW frame at a load rate of 0.07 kN/s [62]. Some of the specimens were subsequently milled to a fine powder for microscopic analysis, after detaining hydration by soaking for 7 d in 1:10 volume isopropanol [63] and vacuum drying to a constant weight.

CSA mineralogical phases were determined on a Bruker D8 Advance X-ray diffractometer (Bruker Corporation, Madrid, Spain) at the following instrumental settings: Cu Kα_1,2_ radiation, 1540 Å, 1544 Å; configuration, no monochromator; and goniometer radius, 217.5 mm. The conditions for anhydrous cement scanning for subsequent Rietveld refinement were: 0.5° fixed divergence slit; 2θ angle range, 5° to 70°; step time, 2 s; step size, 0.02°. The recording conditions for the pastes, using the same instrument, were: variable 6-mm divergence slit; 2θ angle 5° to 60°; step time, 0.5 s; step size, 0.02°. 

Microstructure was analyzed on pellets prepared with 300 mg KBr and 1.0 mg of problem sample and scanned with a Thermo Scientific Nicolet 6700 Fourier transform infrared spectrophotometer covering a frequency range of 4000 to 400 cm^−1^. The spectra acquired with 64 scans at a spectral resolution of 4 cm^−1^ were deconvoluted in the 1350 to 700 cm^−1^ range by fitting to the theoretical curve for the original spectrum (regression coefficient = 1). The second derivative method was applied to identify the position of the bands overlapping on the spectra [64].

Scanning electron microscopic (SEM) studies were conducted on carbon-coated, vacuum-dried 28-d specimens with a Hitachi S-4800 microscope featuring a maximum resolution of 1.4 nm and fitted with a Bruker XRD detector (Billerica, MA, USA). The data were processed with Quantax 400 software.

The heat flow and total heat released associated with the hydration reactions were found with isothermal conduction calorimetry as per standard American Society for Testing and Materials—ASTM C1702 [65] on a Thermometric TAM AIR calorimeter (New Castle, PA, USA) at a constant 25 °C. Pastes containing 10 g of solid and 5 g of distilled water were mixed at a low speed (200 rpm) for 30 s and high speed (800 rpm) for up to 3 min, after which 7.5 g were weighed and placed in the calorimeter.

## 5. Conclusions

The ettringite and AH_3_ formation attendant upon CSA hydration (REF) consumed ye’elimite and anhydrite. Due to the use of a lower-than-stoichiometric w/c ratio (0.5), the ettringite formed was scantly crystalline and the matrix generated was dense. Typical ettringite needles were observed only inside the pores, which afforded sufficient space for crystal growth.

The presence of 5% alkaline salts impacted process kinetics, the reaction products formed and strength development, although these effects varied with the type of salt.

– The use of sodium sulfate had adverse effects. Whilst its presence hastened the initial ye’elimite reaction, raising 2-d strength, at later ages the excess sulfates and alkalis induced thenardite re-precipitation, ettringite carbonation, and gypsum formation. The presence of those minerals in the hardened matrix generated stresses that led to material cracking.
– The addition of sodium carbonate appeared to have no significant effect. Its presence initially induced false setting which after remixing delayed setting times relative to the reference. This was nonetheless the system with the closest resemblance to the reference, possibly because part of the carbonate forming was released in the form of efflorescence. Since thenardite was observed to form at the latest age studied here, however, research on >90-d materials would be advisable.
– Adding sodium silicate had a beneficial effect, hastening hydration and raising mechanical strength relative to the reference at all of the ages studied, 90 d in particular. The presence of this salt catalyzed ye’elimite hydration, raised the amount of ettringite and induced minor strätlingite formation, yielding denser, stronger matrices.

## Figures and Tables

**Table 1:** Isothermal conduction calorimetry parameter values.

Calorimetric Parameter	REF	5NS	5NSi	5NC
Tp (h) ^1^	1.67; 3.41; 6.10	2.52	1.35	2.87; 6.10
Vp (J/g) ^2^	54.02; 103.87;162.8	120.3	74.9	91.7; 184.27
Heat released (J/g), 90 h	305.4	301.9	299.8	293.1

^1^ Tp: peak time. ^2^ Vp: maximum peak intensity.

**Table 2:** FTIR spectrum band positions and areas (in per cent of total) of the reaction products in all systems at all ages analyzed.

		REF								5NS							
		0 d		2 d		28 d		90 d		0 d		2 d		28 d		90 d	
		W ^1^	A ^2^	W	A	W	A	W	A	W	A	W	A	W	A	W	A
AlO_4_ stretching		811	5.56							811	5.56						
		845	0.96							845	0.96						
			-	854	2.88	850	3.28	859	3.74	-	-	850	1.59	851	2.29	857	4.21
		880	19.91	880	7.51	880	4.15			882	19.91	879	4.67				
CO_3_^2−^ bending			-					877	1.59		-			875	1.2	875	1.86
Al-O-H bending			-								-						
			-	1025	2.95	1025	3.32	1029	10.71		-	1024	6.49	1022	6.6	1026	7.36
SiO_4_ asymmetric stretching		911	10.42	914	4.78	915	4.71	910	2.51	911	10.42	921	6.33	914	5.78	914	5.04
		989	5.46	989	3.64	989	3.4	994	3.15	989	5.46	986	3.05	983	3.01	985	2.81
SO_4_^2^ asymmetric stretching		1048	0.2							1048	0.2						
		1100	20.94	1092	14.40	1090	13.61	1088	13.23	1100	20.94	1092	10.07	1091	8.35	1092	7.72
			-	1115	34.67	1114	49.22	1116	49.34		-	1119	39.36	1117	44.33	1116	42.72
		1120	2.85							1120	2.85						
		1150	19.09	1147	17.98	1150	10.47	1149	8.35	1150	19.09	1145	15.45	1142	17.29	1148	17.88
		1175	4.11	1175	2.61	1175	2.6	1175	2.29	1175	4.11	1175	3.11				
			-								-			1155	1.23	1155	1.93
		1190	10.5	1189	8.58	1190	5.24	1190	5.08	1190	10.5	1190	9.88	1180	9.77	1186	8.47
		**5NC**								**5NSi**							
		**0 d**		**2 d**		**28 d**		**90 d**		**0 d**		**2 d**		**28 d**		**90 d**	
		**W**	**A**	**W**	**A**	**W**	**A**	**W**	**A**	**W**	**A**	**W**	**A**	**W**	**A**	**W**	**A**
AlO_4_ stretching		811	5.56							811	5.56						
		845	0.96							845	0.96						
			-	850	3.61	850	3.98	859	4.68			850	2.65	850	3.21	850	3.42
		882	19.91	880	6.12	880	4.94	880	0.64							965	2.08
										882	19.91	880	4.14	880	4.05	880	3.79
CO_3_^2^ bending				875	0.72	875	0.78	875	1.37			875	0.33	876	0.81	876	1.04
Al-O-H bending																	
				1025	1.93	1025	9.28	1022	10.28			1033	3.07	1025	5.24	1025	5.42
		911	10.42	915	4.56	921	2.95	915	2.09	911	10.42	916	4.64	922	4.17	922	3.22
SiO_4_ asymmetric stretching		999	5.46	989	4.38	989	3.54	984	2.58	999	5.46	990	5.24	987	5.2	989	4.15
SO_4_^2^ asymmetric stretching		1048	0.2							1048	0.2						
		1100	20.94	1090	17.95	1091	10.71	1090	9.66	1100	20.94	1090	11.17	1090	8.84	1090	6.25
			-	1118	36.68	1118	44.03	1118	53.18		-	1118	47.42	1118	53.79	1118	57.05
		1120	2.85							1120	2.85						
		1150	19.09	1150	11.58	1150	10.92	1145	7.06	1150	19.09	1150	8.91	1150	7.5	1150	7.26
		1175	4.11	1175	1.67					1175	4.11	1175	2.9				
			-					1155	1.11		-						
		1190	10.5	1190	10.79	1180	8.87	1180	7.35	1190	10.5	1180	9.53	1180	7.19	1190	6.32
Legend:	C_3_A	Ye’elimite		Belite		Anhydrite		Thenardite		AH_3_		Ettringite		Calcite		Strätlingite	

^1^ Wavenumber in cm^−1^; ^2^ Area in percentage of total deconvolution.

**Table 3:** CSA chemical and physical composition and mineralogical phases.

Chemical Composition											
Oxide	CaO	Al_2_O_3_	SO_3_	SiO_2_	MgO	Fe_2_O_3_	Na_2_O	SrO	K_2_O	Others	LoI ^1^
(wt.%)	41.5	23.2	18.36	8.14	3.22	1.05	0.86	0.45	0.44	1.33	1.45
**Mineralogical Phase**											
Phase		C_4_A_3_$	C$	C_2_S	C_3_A		Bredigite	MgO		Gehlenite	
(wt.%)		49.4	23.0	14.6	1.8		6.4	2.7		2.1	
**Physical Property**					**Diameter**						
Blaine (m^2^/kg)		474.6			D10 (μm)			1.06			
BET (m^2^/kg)		1373			D50 (μm)			4.12			
Density (g/cm^3^)		2.90			D90 (μm)			11.51			

^1^ Loss on ignition.

**Table 4:** Material nomenclature and pastes composition.

Sample	CSA (wt.%)	H_2_O (wt.%)	Solid Activator (wt.%)			w/b	%Na_2_O	%Anion
			Na_2_SO_4_	Na_2_CO_3_	Sodium Disilicate ¹			
REF	100	45				0.45	-	-
5NS	95		5				1.51	2.33
5NC				5			2.02	1.95
5NSi					5		0.98	1.90

^1^ Silicate sourced from 2SiO_2_·1Na_2_O·2H_2_O.

## Data Availability

The data presented in this study are available in Supplementary Materials.

## Supplementary Materials

The following are available online, Figure S1. Twenty-eight-day air-cured specimens: (a) REF; (b) 5NS; (c) 5NC; (d) 5NSi.; Figure S2. FTIR spectrum for efflorescence on 28-d 5NC pastes.

## Sample Availability

Not available.
