# Supplementary material for: Effect of Alkaline Salts on Calcium Sulfoaluminate Cement Hydration
Source: Molecules. 2021 Mar 30;26(7):1938. doi: 10.3390/molecules26071938 (PMC8037319; doi:10.3390/molecules26071938)
Supplement: Supplementary file 1 [file molecules-26-01938-s001.pdf]

molecules

Supplementary material

Effect of Alkaline Salts on Calcium Sulfoaluminate Cement Hydration

Luís Urbano D. Tambara Júnior [1,2,*], Janaíde C. Rocha [1], Malik Cheriaf [1], Pilar Padilla-Encinas [2], Ana Fernández-Jiménez [2] and Angel Palomo [2,*]

1 Laboratory of Waste Valorization and Sustainable Materials (ValoRes), Department of Civil Engineering, Federal University of Santa Catarina (UFSC), Florianópolis 88040900, Brazil; janaide.rocha@ufsc.br (J.C.R.); malik.cheriaf@gmail.com (M.C.)
2 Eduardo Torroja Institute, National Research Council (CSIC), 28033 Madrid, Spain; maria.padilla@ietcc.csic.es (P.P.-E.); anafj@ietcc.csic.es (A.F.-J.)
* Correspondence: luistambara@gmail.com (L.U.D.T.J.); palomo@ietcc.csic.es (A.P.)

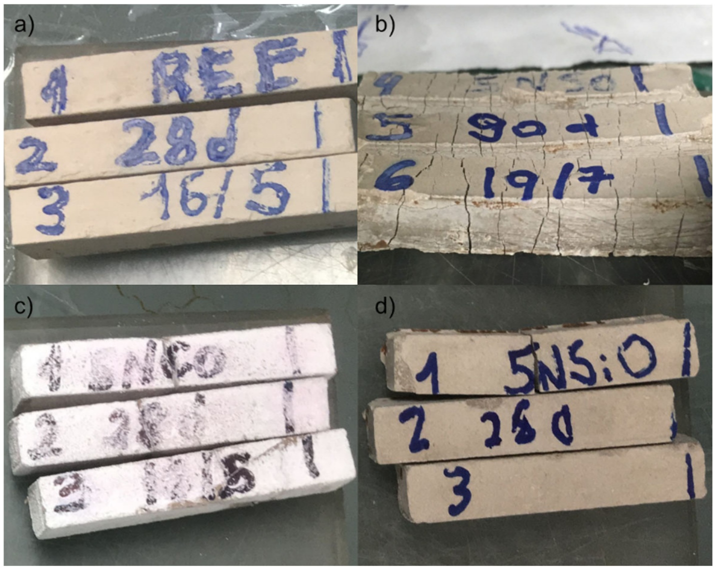

Figure S1. Twenty-eight-day air-cured specimens: **a**) REF; **b**) 5NS; **c**) 5NC; **d**) 5NSi.

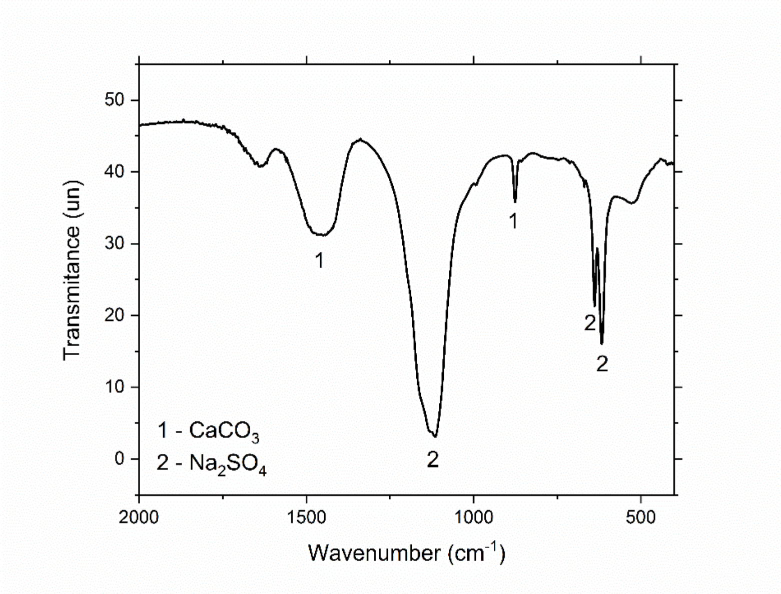

Figure S2. FTIR spectrum for efflorescence on 28 d 5NC pastes.
